# Electronic Health Record-Based Screening for Substance Abuse

**DOI:** 10.1089/big.2018.0002

**Published:** 2018-09-19

**Authors:** Farrokh Alemi, Sanja Avramovic, Mark D. Schwartz

**Affiliations:** ^1^ Health Informatics Program, Department of Health Administration and Policy, George Mason University, Fairfax, Virginia.; ^2^ Department of Population Health, New York University School of Medicine, New York, New York.

**Keywords:** opioid abuse, predictive models, prescription abuse, screening, substance abuse, veterans

## Abstract

Existing methods of screening for substance abuse (standardized questionnaires or clinician's simply asking) have proven difficult to initiate and maintain in primary care settings. This article reports on how predictive modeling can be used to screen for substance abuse using extant data in electronic health records (EHRs). We relied on data available through Veterans Affairs Informatics and Computing Infrastructure (VINCI) for the years 2006 through 2016. We focused on 4,681,809 veterans who had at least two primary care visits; 829,827 of whom had a hospitalization. Data included 699 million outpatient and 17 million inpatient records. The dependent variable was substance abuse as identified from 89 diagnostic codes using the Agency for Healthcare Quality and Research classification of diseases. In addition, we included the diagnostic codes used for identification of prescription abuse. The independent variables were 10,292 inpatient and 13,512 outpatient diagnoses, plus 71 dummy variables measuring age at different years between 20 and 90 years. A modified naive Bayes model was used to aggregate the risk across predictors. The accuracy of the predictions was examined using area under the receiver operating characteristic (AROC) curve in 20% of data, randomly set aside for the evaluation. Many physical/mental illnesses were associated with substance abuse. These associations supported findings reported in the literature regarding the impact of substance abuse on various diseases and vice versa. In randomly set-aside validation data, the model accurately predicted substance abuse for inpatient (AROC = 0.884), outpatient (AROC = 0.825), and combined inpatient and outpatient (AROC = 0.840) data. If one excludes information available after substance abuse is known, the cross-validated AROC remained high, 0.822 for inpatient and 0.817 for outpatient data. Data within EHRs can be used to detect existing or predict potential future substance abuse.

## Introduction

This article reports on the accuracy of using data in electronic health records (EHRs) to screen for substance abuse. To date, substance abuse screening has been based on two approaches. In the first method, the patients are asked to report their drug use, usually through a standardized questionnaire.^1^ This approach has had limited success. Few are surveyed and among those surveyed many deny that their substance use is problematic or should be reported to their primary care clinician. For example, nearly 50% of patients with injection drug use addiction are not identified until they report hepatitis C virus.^2^ A second strategy is to train primary care providers to ask their patients. Among trained primary care providers, 68% reported that they regularly ask new outpatients about drug use^3^; data are not available on what percent ask their current patients, but it is generally assumed to be significantly lower. Neither procedure is widely used; the majority of drug abusers (89.1%) go untreated even though they regularly see their primary care physician.^4^ Given the consequences of continued drug abuse, including dependence,^5,6^ involvement in criminal justice system,^7^ and mortality from overdose or from diseases associated with drug use,^8^ it is important to find an alternative solution that can be easily used in primary care settings. Screening using predictive models is one such alternative that can be used within primary care clinic's EHR systems.

In predictive modeling, the patient's medical history, as captured in the EHR, is used to predict the probability of substance abuse. One advantage of the proposed system of screening is that it does not disrupt clinical processes. The approach relies on neither the efforts of the patient nor the clinician. The computer automatically screens the patient using the data already in the EHRs. Because the information is already available, no new data are collected and current and first-time patients can be screened with little additional effort by the clinicians or patients. Clinicians can then focus their attention on screen-positive patients, a smaller subset than all patients.

There are also other advantages to EHR-based screening. Because predictive modeling is contained entirely within an existing electronic medical record (no data are reported outside the record), there may be limited privacy or security concerns. The proposed approach does not violate HIPAA rules because these rules do not apply to clinical interactions. It also does not violate rules covering disclosure of substance abuse to third parties because no third party is involved. It requires no institutional review board (IRB) review, as it is an operational improvement and not an experimental service that covers some, but not all, patients. It requires no consent as patients provide their medical information in hope that their clinician could manage their care more effectively. True, they may not have explicitly known that their medical history may be used to detect substance abuse, but asking for clinical help implicitly carries the permission to use medical history to diagnose any patient condition, including substance abuse. Nevertheless, since neither the patients nor the clinicians have given explicit consent to the activity, some concerns remain. IRBs and the legal system may not agree with our view that the proposed EHR-based screening does not violate patients' rights to privacy, if it is entirely contained in the medical record.

Given the wide variety of information kept in EHRs (diagnoses, medications, clinical notes, laboratory tests, consults, etc.), there are many ways to construct a predictive model. Some investigators have examined excess opioid prescriptions.^9^ This approach has the advantage of directly measuring exposure risks. We set two goals for our choice of variables to include in the predictive model. First, and of course, it needs to lead to accurate predictions. Second, it needs to shed light on how substance abuse and illness are interconnected. An accurate predictive model is of little use if it does not eventually lead to change in patient's behavior. A focus on the link between illness and substance abuse may frame the issues in ways that produce less defensive posturing and more action by the patient. It may also put substance abuse in the framework of types of diseases typically addressed by primary care providers. Therefore, in this article, we focus on predicting substance abuse from diagnoses.

## Methods

We relied on data available through Veterans Affairs Informatics and Computing Infrastructure (VINCI) for the years 2006 through 2016. We removed duplicated records (same diagnosis occurring for the same patient at the same time) and errors in data entry (patients who had visits before birth and patients who had a visit after date of death). We focused on 4,681,809 unique veterans, 829,827 of whom had a hospitalization. They had 699,754,943 outpatient visits/diagnoses and 17,442,656 hospital diagnoses, 5–15 diagnoses per hospitalization.

### Dependent variable

The dependent variable was substance abuse as identified from 89 diagnoses (e.g., International Classification of Diseases Version 9, ICD9, code 304.00, “opioid-type dependence, unspecified”) using the codes suggested by the Agency for Healthcare Quality and Research.^10^ In addition, we included the diagnostic codes used for identification of prescription abuse.^11^

### Independent variables

The independent variables did not include any of the diagnoses used as the dependent variable. The independent variables included the following:

- Age. This variable was measured by the average of difference of year of encounters and year of birth. Each year between 20 and 90 years was treated as a separate predictor yielding 71 predictors.
- Hospital diagnoses and their repetitions for the same person. Some patients were hospitalized for the same diagnosis repeatedly. We calculated different likelihood ratios for substance abuse for each diagnosis and its repetitions from 1 (no repetition) to more than 5 repetitions. There were 10,540 distinct hospital ICD9 codes. There were 24,140 distinct combinations of diagnoses and repetitions.
- Outpatient diagnoses and their repetitions. We calculated different likelihood ratios for substance abuse for each outpatient diagnosis and its repetitions from 1 (no repetition) to more than 5 repetitions. There were a total of 55,538 outpatient, or repeated outpatient, diagnoses.

In this analysis, substance abuse may have occurred prior, concurrent, or after the time of independent predictors. In general, statisticians encourage measurement of dependent variables after independent variables. This timing recommendation is based on the fact that one does not want to predict something that is already known. While this advice makes sense in many settings, it is not appropriate in screening for substance abuse. Many patients deny their abuse, necessitating the need to detect if substance abuse has already occurred (in short, analysis needs to predict the past).

The model developed here was built on association between diseases and substance abuse. We do not claim causal relationship between diseases and substance abuse. Causal analysis, while of interest in explaining the predictions, is not central to detecting an existing or predicting a future risk of substance abuse. Like most statistical models, such as regression, our predictions are based on the association between disease and substance abuse.

In association studies, both consequences and precedence of substance abuse are reported. In an online file (available through persistent uniform resource locator: https://doi.org/10.15139/S3/1HIMHA), for each diagnosis, we report average number of days free of substance abuse. Sometimes, these days are positive, indicating a predictor that occurs before substance abuse. For other diagnoses, these averages are negative suggesting that the diagnosis may be a consequence of substance abuse.

The reliance on association between diagnoses and substance abuse, and the inclusion of consequences of substance abuse as a predictor, raises the possibility that some predictors may occur after patient's substance abuse is known to the clinician. In these situations, one is predicting a known event, which is not reasonable and cyclical in logic. No prediction is needed, when we know that the patient has had substance abuse diagnoses. To avoid these situations, we excluded from the analysis all predictors that occur after first known diagnosis of substance abuse. The consequences of substance abuse remain in the model for patients who are not diagnosed with substance abuse. In these situations, the detection of substance abuse may identify patients who are denying their substance abuse.

Some of the diagnoses we have included in our analysis may be obvious predictors of substance abuse. For example, inpatient diagnosis of “Suicide by drug or medicine, E950.4” is obviously linked to substance abuse. This diagnosis occurs in 892 patients who have reported substance abuse and surprisingly in 210 patients without such reports. It is not a perfect predictor but it increases the odds of substance abuse by 25-fold. In rare situations, when the patient commits suicide by drug or medicine, the prediction is likely to be quite accurate. Since this code is rare, the impact on overall accuracy is very small. Even when thousands of these rare, but obvious, predictors are used, the overall model accuracy improves by less than 1%. We included these obvious predictors because healthcare is fragmented and reports of these obvious predictors may not reach the primary care providers. A primary care provider may miss the hospitalist's diagnosis that was made a year ago. This is more likely to occur for a patient whose care is complex and the primary provider has to review extensive amount of notes in the record.

There is also another and perhaps more practical reason. Clinicians distrust a computer that makes obvious errors—even if such errors are rare and not statistically significant. Even one drastic and obvious error may lead to the clinician abandoning future alerts. Inclusion of obvious predictors reduces the frequency of embarrassing situations where the computer is way off the mark.

### Methods of prediction

For each diagnosis and its repetition, referred to as predictor, we calculated a likelihood ratio using the prevalence of the predictor among patients with and without substance abuse:

\begin{align*}
 { { \rm { L } } _ { { \rm { Diagnosis } } } } = { \frac { { \rm { Prevalence \;of \;predictor \;among \;known \;substance \;abusers } } }  { { \rm { Prevalence \;of \;predictor \;among \;patients \;with \;no \;indication \;of \;substance \;abuse } } } } .
\end{align*}

A ratio of 2 indicates that the predictor doubles the risk of substance abuse. A ratio of 0.5 indicates that the odds of substance abuse are reduced by half. For each year of age and for each combination of diagnosis and repetition of the diagnosis, a separate likelihood ratio was calculated. These likelihood ratios are available in the online file (to access these data use persistent uniform resource locator: https://doi.org/10.15139/S3/1HIMHA).

We used a variation of naive Bayes to predict from the patient's diagnoses and age the change in odds of substance abuse. It assumes that various predictors are independent from each other. Using the Bayes formula and assumption of independence, the change in odds of substance abuse was calculated from the product of the likelihood ratios:

\begin{align*}
{ \rm{Change \;in \;odds}} = \mathop \prod \limits_{ \begin{matrix} {All \;Patients    \prime } \\ { \;Diagnoses} \\ \end{matrix} } {{ \rm{L}}_{{ \rm{Diagnosis}}}}.
\end{align*}

This method of prediction is similar to the time-varying hazard model^12,13^ in the sense that as patients have new encounters, then new predictions are made: over time, the encounters and the predictions change.

In clinics and in hospitals, the same diagnosis indicates different levels of severity of illness, treatment, and outcomes. For this reason, we built different models for inpatient and outpatient data. When inpatient data are available, the inpatient model can be used. For patients not hospitalized, outpatient data are used. If the combined data are available, then predictions can be made using a noisy OR combination of the two^14^:

\begin{align*}
{ \rm{Overall}} = 1 - \left( {1 - {{{c_{in} \;Inpatient}} \over {1 + {c_{in}\;Inpatient} }}} \right) \left( {1 - {{{c_{out}\; Outpatient}} \over {1 + {c_{out}\; Outpatient} }}} \right)
\end{align*}

In this equation, the constant ${c_{in}}$ is the prior odds of substance abuse in inpatient data; Inpatient is the change in odds of substance abuse using inpatient data; constant ${ c_{out}}$ is the prior odds of substance abuse in outpatient data; and Outpatient is changes in odds of substance abuse using outpatient data.

Other methods of data mining (e.g., Random Forest, Decision Trees) could have been used. We chose naive Bayes because (1) in sparse massive data, such as ours, it is surprisingly accurate^15–26^; (2) it is computationally efficient as assumption of independence allows us to calculate likelihood ratios in smaller subsets of data^27^; and (3) it clarifies the impact of each physical illness on substance abuse, an important goal of our study that could assist in explaining the predictions to the clinicians/patients. Our own experience to date had also indicated that naive Bayes was a sufficiently accurate and robust predictor of outcomes.^28–31^

### Methods of testing accuracy

We randomly selected and set aside 20% of the data for validation. The accuracy of predictions was tested in the validation set. The accuracy was reported using area under the receiver operating characteristic (AROC) curves.^32^

## Results

The average age for the study cohort was 59.45 years. The majority of patients were male (92.1%) and white (73.4%). Because we included all diagnoses as separate predictors, the discussion of the findings is organized into seven different categories that are supported by the literature:

1. Pain-related diagnoses: In some diagnoses, patients receive prescription pain medications, and this could lead to subsequent substance abuse.^33^ In our data, we found many diagnoses that supported such a mechanism. For example, patients with “code 718.25 pathological dislocation of joint, pelvic region, and thigh,” often receive pain medications and had three times higher risk of substance abuse.
2. Diagnoses codes related to socioeconomic factors: It is well known that unemployment, homelessness,^34–37^ and school dropout^38^ are associated with substance abuse. It is less known that diagnoses codes capture some of these socio/economic variables. Our data support the influence of socioeconomic factors. The likelihood ratio associated with the fifth repeated outpatient diagnosis code V60.2 “Economic Problem” increased risk of substance abuse by 12-fold. Fifth repeated hospitalization with diagnosis code V60.0 “Lack of Housing” increased the odds of substance abuse 15 times. First hospitalization with diagnostic code V62.5 “Legal Circumstances” increased odds of substance abuse by 11 times.
3. Mental illness diagnoses: Patients who have mental illness, including depression^39^ and a wide range of other mental diseases.^40–45^ Many mental health diagnoses were associated with substance abuse. For example, second hospitalization for major depression (code 296.20) increased odds of substance abuse by fourfold. Of particular interest are mood disorders occurring within other major diseases (e.g., cerebrovascular disease). Here again mood disorders increased risk of substance abuse. In patients with a fourth hospitalization due to mood disorders occurring in patients with cerebrovascular diseases, the odds of substance abuse increased by threefold. Some diagnoses were highly predictive of substance abuse. For an obvious case, first diagnosis of drug-induced mood disorders (code 292.84) increased risk of substance abuse by 52-fold. First hospitalization for antisocial personality (code 301.7) increased the odds of substance abuse by 31-fold. Of particular interest is the interaction between suicide and substance abuse. Suicide ideation (second outpatient diagnosis code V62.84) increased the odds of substance abuse by 15-fold. These data in general support the finding that a wide variety of mental health illnesses are caused by or lead to substance abuse.
4. Diagnoses patterns indicating weakened immune system: Substance abuse may lead to changes in the body's immune system. This may manifest itself in repeated infections.^46,47^ Injection drug users are more likely to have clostridial infections,^48^ recurrent methicillin-resistant *Staphylococcus aureus* skin and soft-tissue infections,^49^ hepatitis B or hepatitis C,^50^ or sexual infections.^51^ In our data, patients hospitalized five times with “008.45 intestinal infection due to clostridium difficile” were two times more likely to have substance abuse. The literature suggests that patients with recurrent “methicillin-resistant *S. aureus* skin and soft-tissue infections” may have substance abuse^49^; in our data, these patients were more likely to have substance abuse. The data showed that a wide variety of repeated viral infections indicate the presence of substance abuse. Risky practices associated with drug use have been associated with spread of infectious diseases. For example, sex and exchange of needle with infected patients may lead to transmission of HIV, hepatitis B, or hepatitis C^50^ as well as sexual infections.^51^ Our data showed that the third hospitalization for HIV (code 042.) increased odds of substance abuse by sevenfold. The third hospitalization for hepatitis B disease (code 070.32) increased odds of substance abuse by 11 times.
5. Diagnoses measuring physical consequences: Substance abuse affects various body systems, especially the skin, heart, and kidney. Injection drug use can cause localized and systemic effects, including granulomata at the site of injection and in the lungs, including eventual systemic amyloidosis.^52^ In our data, fifth hospitalization with unspecified amyloidosis (code 277.31) increased the odds of hospitalization by twofold. The literature suggests that drug abuse may affect kidney operations. In our data, second hospitalization with “medullary sponge kidney, code 753.17,” increased the odds of substance abuse by 10-fold. Crack cocaine is known to lead to changes in the pulmonary system, including carbon pigmented intra-alveolar macrophages, emphysema, and pulmonary arterial changes; opiates/opioids can lead to pneumonitis and hypoxic brain damage due to their respiratory depressant effects.^52^ In our data, fifth hospitalization with unspecified extrinsic asthma (code 493.00) increased risk of substance abuse by 14-fold. Poisoning by anticommon cold medications (code E945.7) increased risk of substance abuse by 11-fold. First hospitalization for urinary hesitancy (code 788.64) increased the risk of substance abuse by twofold. In the literature, cocaine and amphetamines have the strongest association with stroke.^53^ Our data suggests that fifth hospitalization due to cerebral aneurysm (code 437.3) increased odds of substance abuse by 10-fold. Injecting heroin is known to lead to heroin-associated nephropathy^50^; in our data, fourth hospitalization for nephropathy (code 583.89) was associated with sixfold increase in odds of substance abuse. Heroin can cause arrhythmias and noncardiac pulmonary edema, and reduced cardiac output.^51^ In our data, hospitalization for upper respiratory inflammation due to fumes and vapors (code 506.2) increased the odds of substance abuse by sixfold.
6. Diagnoses indicting drug seeking behavior: Diagnoses that focus on drug seeking behavior may signal potential drug users. Drug seeking is observed in patients who overstate their pain levels in an effort to receive opioid agents. In our data, the fourth hospitalization mentioning the diagnosis of “V65.2 person feigning illness” was associated with 120 times increase in odds of substance abuse. In addition, the literature includes a discussion of legal drugs as gateways to illegal drugs. In our data, patients with first diagnosis of alcohol abuse (code 305.00) were six times more likely to have other substance abuse. The patients who were hospitalized for the third time for accidental poisoning by alcohol (code E860.0) were 23 times more likely to have substance abuse.
7. Diagnoses indicating self-injury: Injury and poisoning may also indicate potential substance abuse. For example, first “accidental poisoning by tranquilizers, code 980.3,” indicates a twofold increase in odds of substance abuse. “Suicide and self-inflicted injury by specified drugs and medicinal substances, code E950.4,” increased the odds of substance abuse by 119-fold.

To further clarify the relationship between diagnoses and substance abuse, the reader can examine the file provided online (see persistent uniform resource locator: https://doi.org/10.15139/S3/1HIMHA). This file includes the list of the diagnoses, number of days the diagnosis occurred prior or after substance abuse, and the likelihood ratio of substance abuse associated with the diagnoses. In Table 1, we present few of these diagnoses and their relationship to substance abuse. This Table shows a select group of mental illness, external injury diseases, and viral infection diagnoses. Many of the findings are not surprising, substance abuse is associated with hospitalization for mental disorders, including PTSD, antisocial personality disorder, drug-induced mental disorders, alcohol mental disorder, episodic mood disorders, continuous alcohol use, and repeated hospitalization for borderline personality disorders. In addition, drug abuse was associated with a series of self-injuries leading to hospitalization, including suicide by antidepressants. Finally, substance abuse was associated with repeated viral infections. In Table 1, we show how the odds of substance abuse change with various diagnoses, some reducing the odds of substance abuse (shown in Table 1 in first row) and others increasing it slightly and still others having a large impact.

**Table 1.** **Selected inpatient diagnoses and associated change in odds of substance abuse**

*LR*	*Mental health*	*External injuries*	*Infections and parasitic*
<1	5th Vascular dementia, uncomplicated (290.40), 47 days after SA	1st Lumbar spinal cord injury (952.2), 535 days before SA	3rd Actinomycosis NOS (039.9), 292 days before SA
1	1st Schizophrenic disorders, residual type, subchronic (295.61), 1248 days before SA	1st Late effect of injury to peripheral nerve of pelvic girdle and lower limb (907.5), 675 days before SA	2nd Pulmonary mycobacteria (031.0), 204 days before SA
2	1st Undersocialized conduct disorder, unaggressive type, unspecified (312.10), 783 days before SA	2nd Crushing injury finger (927.3), 428 days before SA	3rd Salmonella arthritis (003.23), 332 days after SA
3	5th Other specified pervasive developmental disorders, current or active NEC (299.80), 97 days before SA	1st Late effect of internal injury to intra-abdominal organs (908.1), 11 days before SA	1st Pulmonary tuberculosis, unspecified, NOS (011.90), 336 days before SA
4	1st Major depressive disorder: recurrent—severe without psychotic features (296.23), 403 days before SA	2nd Poisoning by sympathomimetics (971.2), 291 days before SA	1st Pulmonary tuberculosis, NEC-unspecified (011.80), 422 days before SA
5	2nd Trans-sexualism with unspecified sexual history NOS (302.50), 405 days before SA	2nd Erythema [first degree] of face and head NOS, (941.10), 66 days before SA	4th *Escherichia coli* septicemia (038.42), 72 days after SA
6	3rd Adjustment disorder with disturbance of conduct (309.3), 421 days before SA	1st Poisoning by hair treatment drugs (976.4), 773 days before SA	3rd *Helicobacter pylori* infection (041.86), 404 days before SA
7	2nd Psychosexual disorder NOS (302.9), 684 days before SA	1st Blister head (910.2), 668 days before SA	1st Pulmonary tuberculosis, NOS-histologically confirmed (011.95), 685 days before SA
8	2nd Anorexia nervosa (307.1), 304 days before SA	1st Superficial injury of conjunctiva (918.2), 305 days before SA	1st Balantidiasis (007.0), 548 days after SA
9	1st Schizoaffective disorder, subchronic with acute exacerbation (295.73), 474 days before SA	1st Poisoning by corticosteroids (962.0), 121 days before SA	5th Human immunodeficiency virus disease (042.), 449 days before SA
10	1st Manic affective disorder, recurrent episode, severe, specified as with psychotic behavior (296.14), 378 days before SA	1st Superficial injury eye NEC (918.9), 344 days before SA	
11	2nd Sleep arousal disorder (307.46), 334 days before SA	1st Poisoning by fibrinolysis agent (964.4), 16 days before SA	
12	4th Schizoaffective disorder, chronic with acute exacerbation (295.74), 482 days before SA	1st Poisoning by local anti-infectives and anti-inflammatory drugs (976.0), 387 days before SA	
13	3rd Obsessive/compulsive disorder (301.4), 482 days before SA	1st Poisoning by cardiovascular agent NEC (972.9), 99 days before SA	
14	5th Schizoaffective disorder NOS (295.70), 449 days before SA	1st Abrasion foot/toe infected (917.1), 217 days before SA	
15	2nd Alcohol abuse in remission (305.03), 291 days before SA	1st Poisoning by methylphenidate (969.73), 190 days before SA	
16	1st Affective personality NOS (301.10), 602 days before SA	2nd Poisoning by antihypertension agent (972.6), 274 days before SA	
17	2nd Attention deficit disorder with hyperactivity (314.00), 457 days before SA	1st Poisoning by uric acid metabolic (974.7), 46 days before SA	2nd Salmonella enteritis (003.0), 132 days before SA
18	3rd Adjustment disorder with mixed anxiety and depressed mood (309.28), 407 days before SA		
19	5th Adjustment reaction NOS (309.9), 442 days before SA	1st Poisoning by expectorants (975.5), 467 days before SA	
20	4th Alcohol abuse unspecified (305.00), 346 days before SA	2nd Poisoning by serotonin reuptake (969.03), 274 days before SA	

Numbers in parentheses refer to the International Classification of Diseases Code version 9. “SA” refers to first diagnosis of substance abuse. Rounded average of days-to or days-after SA is also reported. “NEC” indicates not elsewhere classified, “NOS” indicates not otherwise specified. Full text of all diagnoses is provided online at https://doi.org/10.15139/S3/1HIMHA.

**FIG. 1.**
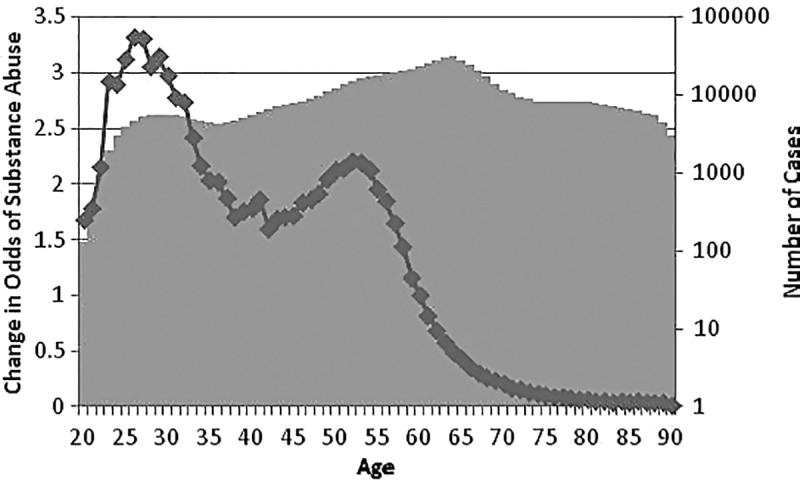
Change in odds of substance abuse depending on age of the hospitalized patients.

The relationship between age of the patient and change in odds of substance abuse is shown in Figure 1. The gray background shows the number of cases on a logarithm scale. Note that for each age group there were sufficiently large numbers of patients to accurately estimate their rate of drug use. The relationship between age and substance abuse was not linear. It peaked in the 20 seconds and declined thereafter with an additional local peak in the midfifties. These two peaks may be associated with use of different substances. In particular, the second peak may be associated with prescription abuse, while the first peak may be associated with illegal drugs. Aside from the two peaks, another feature of interest in these data is the rapid decline in reported substance abuse among patients 75 years or older. Knowing that a patient is older than 75 years reduced the odds of substance abuse.

**FIG. 2.**
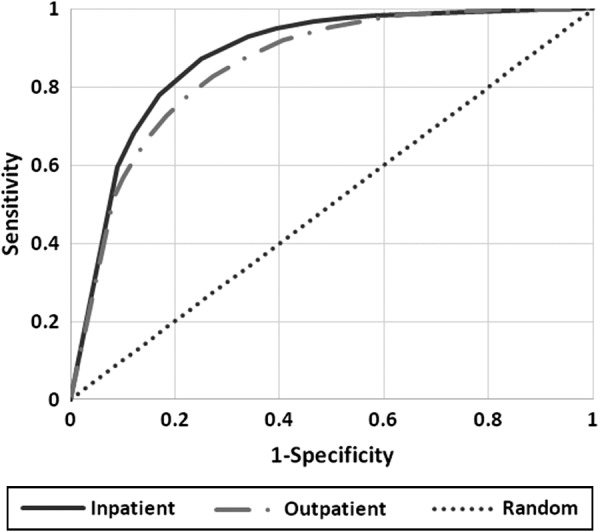
Accuracy of predictive model for substance abuse.

The accuracy of predictions can be assessed using the receiver operating curve (Fig. 2). In receiver operating curves, the dashed, straight, gray line indicates a random prediction and the distance of the operating curves from the dashed line indicates the accuracy of the prediction. Figure 2 shows a receiver operating curve for inpatient and outpatient data using the validation data set. The predictions are slightly more accurate for hospitalized patients. The AROC indicates the extent of accuracy of the predictions. In set-aside validation data, the EHR-based screening accurately predicted substance abuse for inpatient (AROC = 0.884) and outpatient (AROC = 0.825) data. The combined accuracy of both inpatient and outpatient data had AROC of 0.840, indicating that no additional information is gained by adding these two sets of data together. After excluding diagnoses that occurred after known substance abuse, the cross-validated AROC was 0.822 for inpatient and 0.817 for outpatient data. These data suggest relatively low false positive rates and therefore indicate that the screening may be useful in a clinical setting.

## Limitations

The current study did not develop separate predictive models for different substances. Substance abuse may involve many different types of substances (cocaine, heroin, opioid prescription abuse, etc.) that may have distinctly different consequences and may be associated with different illnesses. By including all substances together, we may have reduced the accuracy of our predictions. While the current study makes sense as a first step, future research should distinguish among different types of substance abuse.

The current study focused on patients who utilize the Veterans Administration medical system. This is not only a subset of U.S. population but also a subset of all veterans. It may be problematic to generalize from this study to nonveteran populations. Veterans are predominantly male, older, and have multiple comorbidities.^54–56^ Veterans have higher reported substance abuse.^57^ Therefore, predictions among veterans may be more accurate than predictions in nonveteran groups. It is important to investigate if findings of the current study can be replicated in nonveteran groups. One contribution of this article has been to identify the association between various diseases and substance abuse. We are not aware of any theoretical reason as to why this association should not also occur in the general population. We hypothesize that veterans and nonveterans differ in their reported comorbidities, but the association between comorbidities and substance abuse is the same across the two groups. Additional tests are needed to clarify whether the current method can be applied to nonveteran groups.

## Discussion

This study has shown that hospital or outpatient diagnoses can predict substance abuse accurately. We relied on a comprehensive set of diagnoses, including 24,140 distinct repeated diagnoses. We predicted current or future substance abuse in more than 4.8 million patients monitored over a decade. To avoid modeling noise, the data were randomly divided into training and validation set: 80% of data were used to estimate the association between illness and substance abuse, 20% of data were used to test the accuracy of hospital illness in predicting substance abuse. In set-aside validation data, the model was accurate in predicting substance abuse (AROC of 0.884). Excluding all diagnoses that occurred after substance abuse (i.e., diagnoses that might be consequences of substance abuse), the accuracy dropped to 0.822. If only outpatient data were used, the AROC was 0.822 and excluding potential consequences of substance abuse dropped this to 0.817. These levels of accuracy are high and suggest that the proposed EHR-based screening may be appropriate for use in clinical settings.

Perhaps as important as the increased accuracy is the fact that it is relatively easy to implement the proposed method in various clinics, as it requires no new data collection. It does not rely on effort of either the patient or the clinician and therefore can be widely and consistently implemented across clinics. Patients are not asked to complete cumbersome surveys that later must be integrated into the EHR. Clinicians do not need to discuss substance abuse with the vast majority of patients; they can focus on screen-positive cases. For example, the Veterans Administration requires its 150 medical centers to screen for substance abuse at significant cost and with variable participation rates. The use of predictive models can radically reduce these costs and guarantee consistent participation of all primary care providers and their patients. The Veteran Administration has already aggregated the data in a central data warehouse and the additional step to examine substance abuse of these patients can be carried out. Since patients have already consented to the use of their medical information for improvement of their care, no additional consent is necessary, further streamlining the procedures.

It is helpful to compare the reported AROC levels in this study with the AROC levels reported in the literature for other methods. The reader should be aware that comparing AROC levels across studies is dangerous: the authors of other studies have used other inputs, different evaluation designs, smaller or larger data sets, and so on. We present the data from the literature so that the reader can have a perspective on relative accuracy of each method; further studies are needed before one can claim one method is worse than another. The level of accuracy of our proposed EHR-based screening was comparable with accuracy levels accomplished by direct questionnaires. For example, the abbreviated Alcohol Use Disorder Identification Test for Consumption (AUDIT-C) reports AROC of 0.84 for females and 0.80 for males.^58^ Physicians asking their patients about substance use had AROC of 0.74.^59^ Efforts to predict risk of over dose/suicide from EHRs had AROC higher than 0.80,^60^ suggesting that the performance of predictive model in this study is reasonable and at levels that are comparable with other clinical screening efforts.

A number of investigators have tried to predict substance abuse based on logistic regression. For example, in 2015, Hylan et al. examined 2752 chronic noncancer pain patients initiating chronic opioid therapy.^61^ They developed a weighted risk index for predicting substance dependence based on seven factors, including age, gender, race, smoking status, history of a diagnosis of opioid abuse/dependence, history of drug abuse/dependence, mental disorders (depression, bipolar disorder, anxiety, autism, schizophrenia, dementia, other psychosis, and self-inflicted injury), history of alcohol abuse/dependence, and diagnosis of hepatitis C. The cross-validated AROC curve for problem opioid use was 0.739, lower than our proposed method. The Hylan et al. regression model focuses primarily on key mental illnesses and does not make predictions for patients without these features, for example, cancer patients. Our proposed EHR-based screening is more comprehensive; it includes all diagnoses, as opposed to selected mental health diagnoses. This allows the screening tool to be relevant to a wider set of patients.

Obviously, clinicians cannot implement the proposed screening into their practices without the use of computers. No human can keep track of thousands of predictors; a computer is needed to carry out these calculations. Fortunately, widespread use of EHRs makes it possible to do so. Clinicians will use the proposed EHR-based screening system such as any laboratory tests, with the exception that this test is done inside the computer and not within a laboratory. Like a laboratory test, clinicians may not know how the test works and why it works; but the test results are used by the clinicians.

Additional research is needed to clarify the impact of the proposed screening on patients and providers. The main purpose of the screening is to start a conversation between the clinician and the patient. As a consequence of these conversations, the patients may alter their lifestyle and the clinicians may alter their practices. The clinicians may adjust their pain prescription dosage, may add medications designed to prevent abuse of opioid medications, or may decide to examine alternative methods of pain relief. The primary care clinician may also refer the patient to an addiction specialist. The extent to which our proposed EHR-based screening leads to these practice changes was not evaluated in this study and additional data are needed to clarify the impact of EHR-based screening on practice and lifestyle changes.

One issue that remains to be researched is how the computer should communicate with both the patient and the clinician. Computer alerts are often ignored by clinicians due to reminder fatigue. These alerts are likely to be ignored, if the clinician does not understand the computer's predictions. The computer needs to explain the predictions. Without such explanations, clinicians are likely to distrust the predicted probability numbers. At a minimum, the computer needs to list the top reasons for elevated risks scores. These explanations can further assist the clinician in the conversation with the patient. Additional research is needed to understand how to explain the risk rating to the clinician.

In addition, a communication to the patient needs to be designed. Patients typically receive computer alerts through mailed communications (e.g., many patients receive personalized postcards reminding them to seek flu vaccinations). It is not clear how the risk findings should be explained to the patient. It is not clear if the information should be provided while the patient waits in the clinic or mailed to the patient's home. We have hypothesized that emphasizing the link between physical illness and substance abuse is likely to reduce patient's resistance to behavioral changes. They may be less defensive. This hypothesis needs to be tested.

The procedures for sending personalized alerts are well understood. These procedures do not violate existing privacy and security rules. Nevertheless, patients and clinicians may react to computer findings as an intrusion into their lives. These procedures may not meet patients' expectations that they, and not a computer, should decide on what lifestyle information is revealed in their clinic encounters. One can assume that patients want their clinicians to help them. Yet, some do not reveal their substance use. It is possible that the proposed EHR-based screening violates patients' expectations even though it does not violate existing privacy rules. For that matter, EHR-based screening for any diseases (e.g., diabetes) may surprise patients and providers with unreported lifestyle findings. Predictive medicine, in general, seeks cures for diseases that have not occurred, many of which have behavioral causes. The patients' reactions to predictive medicine are not well understood and deserve careful study.

A final point needs to be made about misuse of the proposed system. We have planned this system to be used inside the EHR, for the benefit of the primary care providers and their patients. Others, for example, insurance companies, have access to the medical history of the patient through claims data. Therefore, they could use the system proposed here. If the insurance companies intend to use the proposed system to alert their case managers to assist primary care management of the patient, then the use may make sense. If they intend to use it to restrict access to insurance, then the use of our methods is not appropriate. There are legislations that prohibit use of genetic data to deny access to insurance. There are no such legal restrictions on use of predictive models to deny access to insurance. It may be necessary to put in place such legal protections for EHR-based screening of substance abuse or any other illnesses. We are also concerned with employers using the medical history of their employees for substance abuse screening. Some employees actively screen employees with random urine tests. These employers may find our method simpler and may wish to switch to our method. We designed the proposed predictive model to be used in a collaborative context—where patients and clinicians are working together to improve their health. The use of the proposed system by employers may create situations where patients may not file claims in fear that their substance abuse may be discovered. We call for legislations that prohibit misuse of this and other predictive models. Besides use in primary care encounters, the proposed EHR-based screening can be used to calculate clinic-wide performance measures. Often, insurers and payers compare the performance of healthcare providers. The Veterans Administration, for example, requires each medical center to screen a sample of patients and report the frequency of screening and extent of substance abuse found across all screened patients. These performance reports are time-consuming and must be done regularly. Many Veterans Administration centers fail to comply. Our methods may provide a simpler, and perhaps more accurate, way to report performance of groups of clinicians.

## Abbreviations Used

HER: electronic health records
VINCI: Veterans Affairs Informatics and Computing Infrastructure
PTSD: posttraumatic stress disorder
AROC: area under the receiver operating characteristic
AUDIT-C: Alcohol Use Disorder Identification Test for Consumption
